# Biocontrol Potential of a Mango-Derived *Weissella paramesenteroides* and Its Application in Managing Strawberry Postharvest Disease

**DOI:** 10.3390/jof11070538

**Published:** 2025-07-19

**Authors:** Xiyu Zhang, Bang An

**Affiliations:** 1 School of Breeding and Multiplication (Sanya Institute of Breeding and Multiplication), Hainan University, Sanya 572025, China; 15292586507@163.com; 2 School of Tropical Agriculture and Forestry (School of Agriculture and Rural Affairs & School of Rural Revitalization), Hainan University, Sanya 572025, China

**Keywords:** *Weissella paramesenteroides*, volatile organic compounds (VOCs), antifungal activity, postharvest disease, biological control

## Abstract

Postharvest fungal diseases are a major cause of fruit spoilage and economic losses, particularly in perishable commodities like strawberries. In this study, a plant-derived *Weissella paramesenteroides* strain R2 was isolated from the mango fruit surface and evaluated for its antifungal potential. Dual-culture assays revealed the strong inhibitory activity of strain R2 against key postharvest pathogens, including *Botrytis cinerea*, *Colletotrichum gloeosporioides*, and *Fusarium oxysporum*. Notably, cell-free fermentation broth exhibited no antifungal activity, whereas the volatile organic compounds (VOCs) produced by R2 significantly suppressed fungal growth in sealed plate assays. GC-MS analysis identified 84 VOCs, with pyrazines as the dominant group. Three major compounds, 2,5-dimethylpyrazine, 2,4-di-tert-butylphenol, and 2-furanmethanol, were validated for their antifungal activity. The application of R2 VOCs in strawberry preservation significantly reduced disease incidence and severity during storage. These findings highlight *W. paramesenteroides* R2 as a promising, food-safe biocontrol agent for postharvest disease management via VOC-mediated mechanisms.

## 1. Introduction

Postharvest diseases caused by fungal pathogens pose a major threat to the quality, shelf life, and marketability of fresh fruits and vegetables, leading to substantial economic losses worldwide [1]. This problem is especially severe in tropical and subtropical regions, where high humidity and warm temperatures provide optimal conditions for fungal development. Fruits, such as mangoes, bananas, pineapples, papayas, and strawberries, are highly vulnerable to fungal colonization and decay during storage and transportation [2,3]. Fungi, such as *Botrytis cinerea*, *Penicillium expansum*, *Colletotrichum* spp., and *Rhizopus stolonifera*, are among the most common postharvest pathogens; they can infect fruits through wounds or natural openings and develop rapidly under storage conditions. The adaptability of these fungi, combined with the limited innate immune responses of harvested fruits, makes postharvest disease control particularly challenging [4,5].

Synthetic fungicides have been traditionally used to control postharvest diseases. Common agents such as thiabendazole, imazalil, fludioxonil, and iprodione are widely applied during postharvest handling [6,7]. While effective in suppressing fungal growth, these chemicals are increasingly constrained by several drawbacks. First, chemical residues on fruit surfaces may pose risks to human and animal health and contribute to environmental contamination. Second, the repeated and widespread use of synthetic fungicides has led to the emergence of resistant pathogen strains, reducing their long-term effectiveness [8]. Consequently, many countries have restricted the use of postharvest fungicides to a limited number of approved compounds. These concerns underscore the need for safer and environmentally friendly alternatives for managing postharvest fungal diseases.

Biocontrol, also named biological control, has emerged as a promising strategy to replace or complement chemical fungicides in postharvest disease management [9]. This approach utilizes antagonistic microorganisms or their metabolites to inhibit the growth and pathogenicity of fungal pathogens [10,11]. Over the past two decades, considerable progress has been made in identifying and characterizing a wide range of biocontrol agents. These include bacterial species such as *Bacillus subtilis*, *Pseudomonas fluorescens*, and *Paenibacillus polymyxa* [12]; yeast species such as *Candida oleophila*, *Metschnikowia fructicola*, and *Debaryomyces hansenii* [13]; and filamentous fungi including *Trichoderma harzianum* and *Trichoderma atroviride* [14]. These organisms employ diverse mechanisms of action, such as competition for nutrients and space, production of antifungal metabolites, secretion of hydrolytic enzymes, and induction of host resistance responses [15]. Several microbial products have even been commercialized, such as Serenade^R^ (based on *Bacillus subtilis* strain QST 713) and Shemer^TM^ (based on *Metschnikowia fructicola*), and are already being used effectively to reduce decay in fruits during postharvest storage [16,17].

In recent years, members of the family *Lactobacillaceae*, best known for their probiotic properties, have drawn increasing attention for their antifungal potential. This family includes genera such as *Lactobacillus*, *Weissella*, *Leuconostoc*, and *Pediococcus*, which are commonly found in fermented foods, on plant surfaces, and in the gastrointestinal tracts of animals [18,19]. Members of this group are generally regarded as safe and have a long-standing history of use in the food industry [20]. Beyond their health-promoting benefits, many *Lactobacillaceae* strains are capable of producing antifungal compounds, including organic acids, hydrogen peroxide, bacteriocins, and volatile organic compounds (VOCs) [21]. Among these, VOCs have attracted particular interest for their ability to inhibit fungal growth via gas-phase contact, offering the advantage of non-contact biocontrol [22]. The ability of these bacteria to colonize fruit surfaces and produce bioactive metabolites makes them attractive candidates for postharvest biocontrol applications.

In the present study, we aimed to isolate a food-safe biocontrol agent from a natural fruit source, characterize its antifungal mechanism, and evaluate its practical application in managing postharvest fruit decay. We isolated and functionally characterized a strain designated *Weissella paramesenteroides* strain R2 from the surface of mango fruit on the basis of its strong antifungal activity. A mechanism investigation revealed that strain R2 produces VOCs capable of suppressing fungal growth in a sealed system. To evaluate its applicability, we assessed the effectiveness of *W. paramesenteroides* R2 in controlling postharvest disease development in strawberries. Overall, our findings highlight *W. paramesenteroides* R2 as a promising, underexplored, and safe biocontrol agent for postharvest disease management through VOC-mediated antifungal activity.

## 2. Materials and Methods

### 2.1. Isolation and Identification of Bacteria with Antifungal Activity

Surface epiphytic bacteria were isolated from fresh, healthy mango (*Mangifera indica*) fruits collected from a local orchard. The fruit surfaces were gently rinsed with sterile distilled water, followed by immersion in 0.85% NaCl solution and shaking at 150 rpm for 30 min. The suspension was serially diluted and spread onto MRS (de Man, Rogosa, and Sharpe) agar plates. After incubation at 28 °C for 72 h under aerobic conditions, morphologically distinct colonies were picked and purified.

Bacterial isolates were identified based on 16S rDNA gene sequencing. Genomic DNA was extracted using a commercial bacterial DNA extraction kit (TIANGEN, Beijing, China), and the 16S rRNA gene was amplified using universal primers 27F (5′-AGAGTTTGATCMTGGCTCAG-3′) and 1492R (5′-TACGGYTACCTTGTTACGACTT-3′). PCR products were purified and sequenced, and resulting sequences were compared with entries in the NCBI GenBank database using BLAST at https://blast.ncbi.nlm.nih.gov/Blast.cgi. A phylogenetic tree based on 16S rDNA sequences was constructed using MEGA 11.0 [23].

### 2.2. Screening the In Vitro Antifungal Activity of Bacteria

Antifungal activity of bacterial strains was initially evaluated using a dual-culture assay. The plant pathogenic fungi *B. cinerea*, *C. gloeosporioides*, and *Fusarium oxysporum* f. sp. *cubense* were selected as indicator fungi due to their widespread presence and economic importance in postharvest diseases of many fruits. These fungi represent diverse genera and infection mechanisms, allowing us to assess the broad-spectrum antifungal potential of the candidate biocontrol agent. A 5 mm diameter mycelium plug of each indicator fungus was placed at the center of a 9 cm Petri dish containing potato dextrose agar (PDA). Each bacterial isolate was then streaked in a straight line approximately 3 cm away from the fungal plug. Plates inoculated only with fungal plugs, without any bacterial treatment, served as negative controls (CK). Fungal inhibition was evaluated by measuring the colony growth and comparing with those of the corresponding control plates. Each treatment was performed in triplicate, and the experiments were repeated three times. Among the tested isolates, the strain that exhibited the strongest and most consistent antifungal activity was selected for further study and designated as strain R2.

### 2.3. Evaluation of Antifungal Activity of Fermentation Broth and VOCs Produced by Strain R2

To investigate the antifungal activity of extracellular metabolites and volatile compounds produced by strain R2, two complementary assays were conducted using its cell-free fermentation broth and VOCs. For the fermentation broth assay, strain R2 was cultured in MRS broth at 37 °C for 72 h. The bacterial culture was centrifuged at 8000× *g* for 10 min and filtered through a 0.45 μm membrane to obtain cell-free fermentation broth. This broth was mixed with molten PDA medium at dilution rate of 1:50, 1:100, and 1:500 (*v*/*v*) and poured into Petri dishes. Indicator fungal pathogens were subsequently inoculated onto the surface of the solidified medium, and colony growth was monitored to evaluate antifungal activity. For the VOC assay, a two-sealed-base-plate system was employed, as previously described [18]. Briefly, the bottom half of one Petri dish containing PDA was inoculated with an indicator fungal pathogen, while the bottom half of another Petri dish was filled with MRS agar and inoculated with strain R2. The two dishes were sealed together with parafilm to prevent physical contact between the media and ensure only airborne exchange. Uninoculated MRS plates served as negative controls.

To further validate the antifungal contribution of individual volatile compounds, three major compounds, 2,5-dimethylpyrazine, 2-furanmethanol, and 2,4-di-tert-butylphenol, were tested. For treatment, filter papers were placed inside the bottom halves of Petri dishes and loaded with 50–150 μL of the respective synthetic compounds. Specifically, 2,5-dimethylpyrazine was dissolved in ddH_2_O to a final concentration of 1 mol L^−1^; 2,4-di-tert-butylphenol was dissolved in ethanol to a final concentration of 1 mol L^−1^; and the liquid compound 2-furanmethanol was applied directly without dilution. These dishes were then sealed in a manner similar to the VOC assay described above. All plates were incubated at 28 °C for 5–7 d, and the colony growth was analyzed. Each treatment was performed in triplicate, and the experiments were repeated three times.

### 2.4. Gas Chromatography–Mass Spectrometry (GC-MS) Analysis of VOCs

The VOCs produced by R2 strain R2 were identified using a protocol, as previously described [24]. Briefly, R2 was cultured in 20 mL headspace bottles containing MRS agar medium and incubated for 3 d. To distinguish VOCs derived from bacterial metabolism from those originating in the growth medium, a blank control consisting of uninoculated MRS agar was included and treated under identical conditions. For sampling, 10 μL of 2-octanol (10 mg L^−1^ in double-distilled water) was added to each vial as an internal standard (IS). The headspace air was subsequently analyzed by Biotree Biotech Co., Ltd. (Shanghai, China) using an Agilent 7890B gas chromatograph coupled with a 5977B mass spectrometer (Agilent Technologies, Santa Clara, CA, USA). The VOC separation, mass spectra, and data process were performed as previously described [24]. Raw data were processed using ChromaTOF 4.3X software, and compounds were identified based on spectral matching with the NIST database. Only peaks with a similarity score above 800 were considered as reliably identified compounds. To determine R2-specific VOCs, the profiles of the bacterial samples were compared to the blank control. Compounds that were absent in the control or significantly enriched in the R2 samples (Log_2_FoldChange > 1) were considered as R2-specific VOCs.

### 2.5. Evaluation of Fruit Preservation by R2 VOCs

To assess the effectiveness of R2 in preserving strawberries, fresh strawberries with same ripeness and uniform size were obtained from local orchards. Strawberries were randomly divided into groups and stored in fresh-keeping boxes (approximately 5500 mL volume). For the VOC treatment group, three MRS agar plates inoculated with R2 (pre-incubated at 37 °C for 3 d) were placed in each box without direct contact with the fruit. The control group received uninoculated MRS agar plates under the same conditions. All boxes were sealed and stored at 28 °C with 90% relative humidity for 3 days. After storage, disease severity was evaluated based on visible fungal infection using a four-point disease index scale: 0: no visible disease symptoms; 1: less than 25% of fruit surface covered with mycelium; 2: 25–50% of fruit surface covered with mycelium; 3: more than 50% of fruit surface covered with mycelium. The average disease index was calculated for each group. All treatments were conducted in triplicate, and the entire experiment was repeated three times.

### 2.6. Statistical Analysis

All experiments were performed in triplicate, and the data are presented as mean ± standard deviation (SD). Data were analyzed based on Student’s *t*-test or one-way ANOVA with Duncan’s multiple range test via SPSS 18. Differences at *p* < 0.05 were considered significant.

## 3. Results

### 3.1. In Vitro Antagonistic Activity of R2

**Figure 1:**
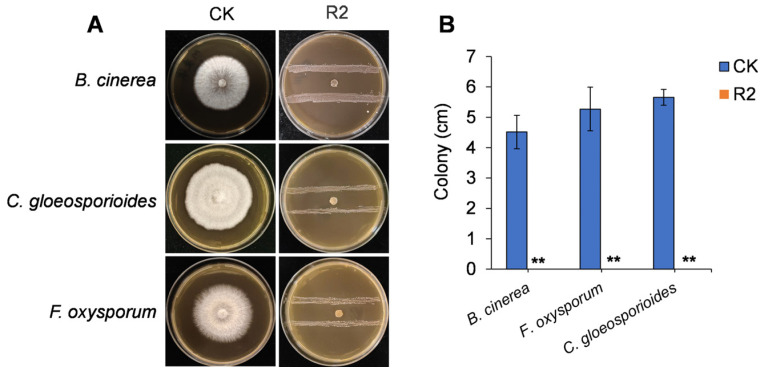
In vitro antagonistic activity of strain R2 against postharvest fungal pathogens. (**A**) Dual-culture assays showing fungal growth inhibition by R2 against *Botrytis cinerea*, *Colletotrichum gloeosporioides*, and *Fusarium oxysporum* on PDA plates after 5 d of incubation at 28 °C. CK: control check. (**B**) Colony diameter in CK and R2-treated plates. Values are shown as the means ± standard deviations (SD). Asterisks indicate significant difference at *p* < 0.01 (**).

To identify bacterial isolates with antifungal potential, a total of 15 strains were obtained from the surface of mango fruits and screened against three common postharvest fungal pathogens: *B. cinerea*, *C. gloeosporioides*, and *F. oxysporum*. Among them, a strain designated R2 exhibited the strongest antagonistic activity in dual-culture assays. As shown in Figure 1A, strain R2 strongly inhibited the growth of all tested pathogens when co-cultured on PDA plates, while normal mycelial growth was observed in the control plates without bacterial inoculation. Quantitative analysis revealed that fungal growth was nearly completely suppressed in the presence of R2 (Figure 1B) at 5 days post-inoculation, suggesting strong and broad-spectrum antifungal activity under in vitro conditions.

### 3.2. Morphological Observation and Molecular Identification of Strain R2

**Figure 2:**
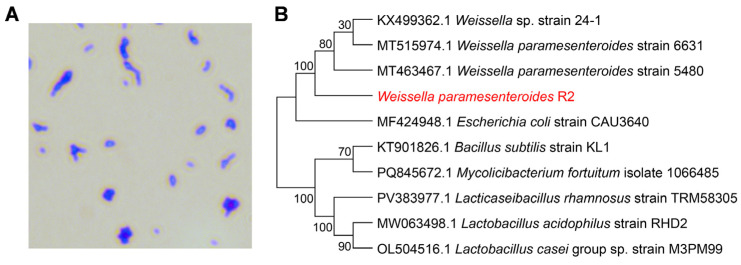
Morphological and molecular identification of strain R2. (**A**) Light microscopy image showing the cell morphology of strain R2 after Gram staining. (**B**) Phylogenetic tree based on 16S rRNA gene sequences using the neighbor-joining method. The red letter indicates *W. paramesenteroides* R2.

Strain R2 was further characterized based on morphological and molecular features. Microscopic examination after crystal violet staining revealed that R2 cells were short rod-shaped bacteria, typically appearing singly or in pairs (Figure 2A). For molecular identification, the 16S rRNA gene of strain R2 was amplified and sequenced. Phylogenetic analysis using the neighbor-joining method showed (Figure 2B) strain R2 clustered closely with *W. paramesenteroides* strains 6631 and 5480. The sequence shared over 99.5% identity with these reference strains, confirming that the isolate belongs to *W. paramesenteroides*. Taken together, these results indicate that the isolate is a member of *W. paramesenteroides*, and it was, therefore, designated *W. paramesenteroides* R2.

### 3.3. Antifungal Activity of VOCs Produced by W. paramesenteroides R2

**Figure 3:**
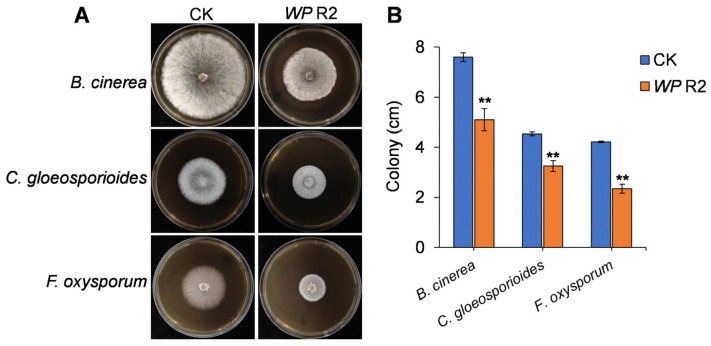
Antifungal activity of VOCs produced by *Weissella paramesenteroides* R2. (**A**) Colony morphology of *B. cinerea*, *C. gloeosporioides*, and *F. oxysporum* on PDA plates after 5 days of exposure to VOCs emitted by strain R2 (WP R2). (**B**) Colony diameter in CK and R2-treated plates. Values are shown as the means ± standard deviations (SD). Asterisks indicate significant difference at *p* < 0.01 (**).

The VOCs released by *W. paramesenteroides* R2 were evaluated for their antifungal activity using a sealed two-plate system. As shown in Figure 3A, VOCs emitted by R2 markedly inhibited the mycelial growth of all three tested fungal pathogens compared to the CK. Quantitative analysis revealed that VOC exposure led to significant reductions in the colony by 33%, 28%, and 44% for *B. cinerea*, *C. gloeosporioides*, and *F. oxysporum*, respectively (Figure 3B). In parallel, the antifungal activity of the cell-free fermentation broth of R2 was also examined. However, no inhibitory effect was observed against any of the tested pathogens at all tested dilutions, suggesting that the antifungal activity of strain R2 is primarily mediated by volatile metabolites rather than soluble secreted compounds.

### 3.4. Identification and Classification of VOCs Produced by W. paramesenteroides R2

**Figure 4:**
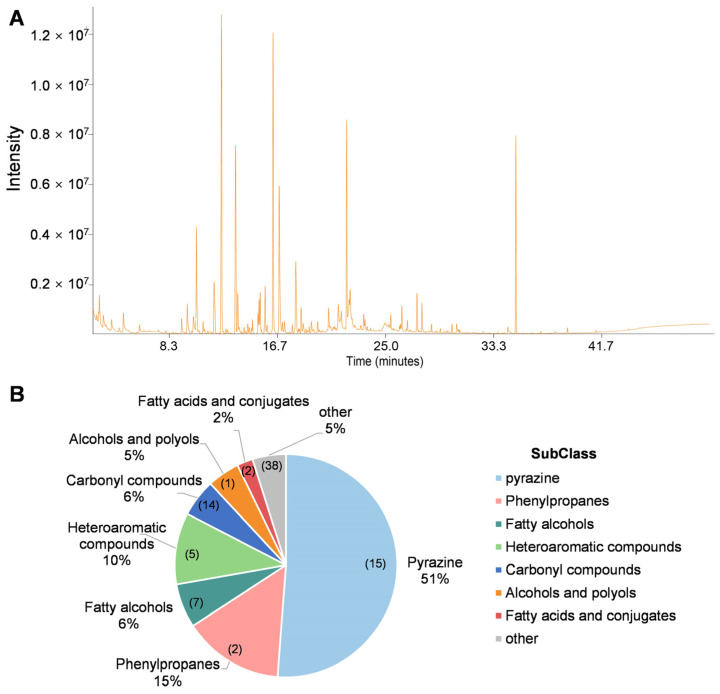
GC-MS analysis of volatile compounds produced by *Weissella paramesenteroides* R2. (**A**) Total ion chromatogram (TIC) of VOCs emitted by strain R2 after 3 d of incubation on MRS agar at 28 °C. (**B**) Classification and relative abundance of 84 identified volatile compounds into major chemical subclasses. The percentages represent the proportion of the substance in the total amount, and the numbers in parentheses indicate the types of substances.

To identify the VOCs responsible for the antifungal activity of strain R2, headspace volatiles were analyzed using gas chromatography–mass spectrometry (GC-MS). The total ion chromatogram (TIC) revealed a complex mixture of VOCs with multiple prominent peaks, indicating the production of a diverse array of metabolites by *W. paramesenteroides* R2 (Figure 4A). In total, 295 volatile compounds were identified, of which 143 compounds with similarity more than 800 were selected for the following analysis (Supplementary Table S1).

The relative contents of these compounds were quantitated by comparing their peak areas to that of the internal standard (IS). Through comparing with the blank control samples, a total of 84 compounds were identified as specifically produced by *W. paramesenteroides* R2. Subsequently, these compounds were classified into eight major chemical subclasses based on structural similarity and functional groups (Figure 4B). Pyrazine derivatives were the most abundant class, accounting for 51% of the total VOC profile. Other prominent groups included phenylpropanes (15%) and heteroaromatic compounds (10%).

### 3.5. Validation of Antifungal Activity of Key VOCs

**Figure 5:**
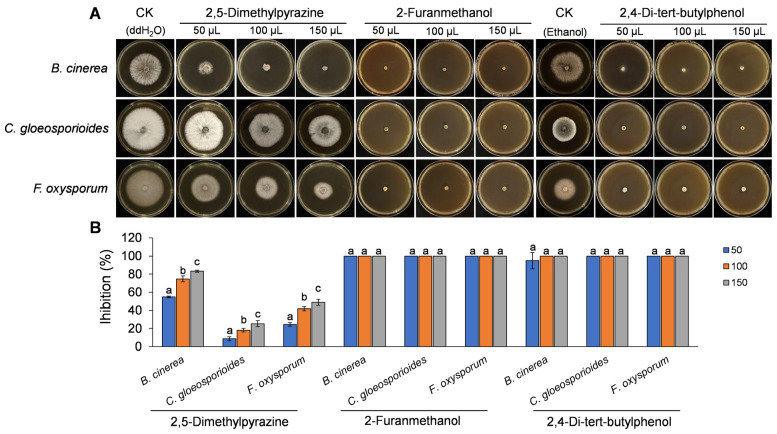
Validation of the antifungal activity of representative VOCs produced by *W. paramesenteroides* R2. (**A**) The inhibitory effects of three synthetic volatile compounds on the growth of *Botrytis cinerea*, *Colletotrichum gloeosporioides*, and *Fusarium oxysporum* in a two-sealed-plate system. Control treatments (CK) were performed using ddH_2_O or ethanol, as appropriate. (**B**) Colony growth inhibition under different concentrations (50, 100, and 150 μL) of each compound. Values are shown as the means ± standard deviations (SD). Different letters indicate significant difference at *p* < 0.05.

To verify the antifungal roles of specific volatiles produced by *W. paramesenteroides* R2, the three most abundant compounds identified in GC-MS analysis (Table 1), including 2,5-dimethylpyrazine, 2-furanmethanol, and 2,4-di-tert-butylphenol, were selected for in vitro validation using a sealed two-plate system. As shown in Figure 5, all three compounds significantly inhibited fungal growth compared to the respective controls (ddH_2_O or ethanol). 2,5-dimethylpyrazine exhibited a dose-dependent inhibitory effect, with maximum inhibition rates reaching approximately 83% for *B. cinerea*, 25% for *C. gloeosporioides*, and 49% for *F. oxysporum* at 150 μL. In contrast, 2-furanmethanol and 2,4-di-tert-butylphenol demonstrated consistently high antifungal efficacy across all tested volumes (50–150 μL), achieving inhibition rates of nearly 100% against all pathogens, with no significant differences among dosages, indicating their potent and broad-spectrum activity. These results confirmed that the identified VOCs contribute directly to the antifungal properties of *W. paramesenteroides* R2.

### 3.6. Application of W. paramesenteroides R2 VOCs for Postharvest Disease Control in Strawberry

**Figure 6:**
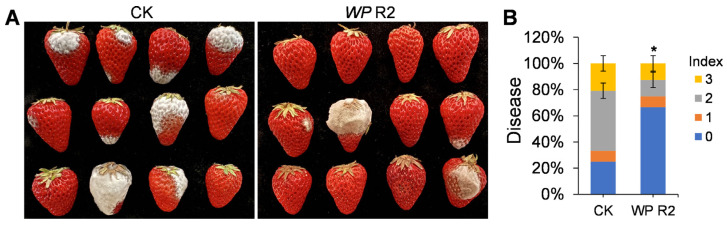
Effect of *W. paramesenteroides* R2 VOCs on controlling postharvest disease in strawberries. (**A**) Disease symptoms of strawberries stored with or without *W. paramesenteroides* R2 VOCs after incubation for 5 d at 25 °C and 90% relative humidity. (**B**) Distribution of disease severity index scores for each group. Values are shown as the means ± standard deviations (SD). Asterisks indicate significant difference at *p* < 0.05.

To evaluate the practical application potential of *W. paramesenteroides* R2 for fruit preservation, it was used for reducing fungal disease in postharvest strawberries. After 5 d of storage at 25 °C and 90% relative humidity, fruits exposed to R2 VOCs exhibited noticeably lower levels of fungal infection compared to untreated controls (Figure 6A). Quantitative assessment using a standardized disease index revealed a significant shift in infection severity between the two groups. In the VOC-treated group, approximately 65% of the fruits remained symptom-free (index 0), compared to only 30% in the control group (Figure 6B). Moreover, the proportion of severely infected fruits (index 3) was substantially lower in the treatment group. These results indicate that VOCs produced by *W. paramesenteroides* R2 can effectively suppress postharvest fungal development in strawberries.

## 4. Discussion

Biocontrol has been researched as an environmentally friendly alternative to chemical fungicides in the management of postharvest diseases [25,26]. Biocontrol agents, particularly those derived from natural microorganisms, have demonstrated great potential due to their environmental compatibility and specificity. Various bacterial and fungal genera have been extensively explored for their antagonistic activity against plant pathogens [9]. However, the biocontrol potential of lactic acid bacteria has remained relatively underexplored.

*W. paramesenteroides*, a heterofermentative lactic acid bacterium in the family *Lactobacillaceae*, is traditionally known for its roles in the fermentation of vegetables, dairy, and meat products [27,28,29]. It is generally regarded as safe and is frequently associated with fermented foods and plant microbiota. Genomic and functional studies have highlighted its enzymatic versatility, ability to produce antimicrobial peptides, and probiotic properties [30,31]. Despite these favorable characteristics, the application of *W. paramesenteroides* in plant disease biocontrol has not been widely investigated, and even fewer studies have examined the mechanisms underlying its antagonistic effects. Although limited studies have reported antifungal activity by *Weissella* species, such as its inhibition against *Aspergillus flavus* growth and aflatoxin production [32], practical use in postharvest disease management remains unverified.

In the present study, we isolated and characterized strain *W. paramesenteroides* R2 from the surface of mango fruit and evaluated its antifungal potential against three major postharvest fungal pathogens: *B. cinerea*, *C. gloeosporioides*, and *F. oxysporum*. In vitro dual-culture assays revealed strong and broad-spectrum antagonistic activity by strain R2 (Figure 1). Notably, the cell-free fermentation broth showed no inhibitory effect, suggesting that non-volatile extracellular metabolites, such as organic acids or bacteriocins, were not the primary antifungal agents. Instead, the antifungal effect was attributed to VOCs emitted by R2, as demonstrated in sealed plate assays (Figure 3). This finding aligns with the growing evidence highlighting the antimicrobial efficacy of VOCs, which can inhibit pathogens through airborne transmission without direct physical contact [33,34].

VOCs have recently emerged as a novel and promising mode of action for biocontrol agents. Several studies have reported the ability of microbial VOCs to inhibit fungal growth, spore germination, and mycotoxin biosynthesis [35,36]. For example, VOCs produced by *Bacillus subtilis* and *Streptomyces* spp. have been shown to suppress a broad range of phytopathogens through the release of compounds [37,38,39]. Similarly, *Pseudomonas fluorescens* and *Metschnikowia pulcherrima* have demonstrated the VOC-mediated inhibition of fungal pathogens on fruit surfaces [40,41]. However, studies on VOCs from lactic acid bacteria, especially *Weissella*, are still limited. Headspace VOCs from strain R2 were analyzed via GC-MS, revealing a complex profile of 84 compounds. Among these, pyrazines were the most abundant class, comprising over 50% of the total identified volatiles (Figure 4; Supplementary Table S1). Pyrazines are nitrogen-containing heterocyclic compounds known for their characteristic aroma and well-documented antimicrobial properties [42]. In our analysis, 2,5-dimethylpyrazine was the dominant compound. This molecule has previously been shown to inhibit several phytopathogenic fungi, including *F. graminearum* and *P. expansum* [43,44,45]. Our functional validation confirmed that this compound significantly inhibited *B. cinerea*, *C. gloeosporioides*, and *F. oxysporum*, with increasing doses correlating with enhanced inhibition (Figure 5). Notably, it displayed a particularly potent effect against *B. cinerea*. In addition, two other major volatiles were identified and validated for antifungal activity: 2,4-Di-tert-butylphenol and 2-furanmethanol. The phenolic compound 2,4-Di-tert-butylphenol has been previously reported to disrupt fungal cell membranes and induce oxidative stress, leading to growth inhibition of *F. oxysporum* and *Ustilaginoidea virens* [46,47]. 2-furanmethanol, also known as furfuryl alcohol, is a furan derivative with antimicrobial and antioxidant properties and is commonly found in fermentation products [48]. Consistent with these reports, our results demonstrated that the two compounds significantly suppressed pathogen growth across all three tested fungi, even at low doses (Figure 5). These findings underscore the role of multiple VOCs in the antifungal activity of strain R2.

To assess the practical application potential of R2, we conducted a fruit preservation assay using strawberries, which are highly susceptible to postharvest fungal decay. The VOCs produced by R2 significantly reduced disease incidence and severity under storage conditions mimicking commercial environments. Approximately 65% of the VOC-treated fruits remained disease-free, in contrast to only 30% in the untreated control. Furthermore, the proportion of severely infected fruits (disease index 3) was markedly reduced under R2 treatment. These results are consistent with previous studies, where VOCs from *Bacillus strains* were successfully applied to reduce fungal diseases in fruits [43,49]. The microbial composition of fruit surfaces is known to vary depending on multiple factors. Native fruit-associated microbiota can contribute to disease resistance by competitively excluding pathogens, modulating host immune responses, and producing antimicrobial metabolites. Notably, the VOCs emitted by *W. paramesenteroides* R2 may not only inhibit fungal pathogens but could also play a role in shaping the fruit microbial community and suppress disease development indirectly by enhancing fruit microbiota [50]. Future studies incorporating microbiome analysis under VOC exposure will be valuable in uncovering this mechanism. Collectively, our findings highlight the practical viability of *W. paramesenteroides* R2 as a non-contact biocontrol agent for postharvest applications.

In conclusion, this study is the first to demonstrate that *W. paramesenteroides* R2 can function as an effective biocontrol agent against postharvest pathogens via the emission of antifungal VOCs. The identification and validation of specific inhibitory compounds, including 2,5-dimethylpyrazine and 2,4-di-tert-butylphenol, provide a mechanistic basis for its action and pave the way for the future development of natural, food-safe antifungal strategies in postharvest disease management.

## Figures and Tables

**Table 1:** The top 10 identified compounds in the VOCs of *W. paramesenteroides* R2.

Number	Name	Chemical Subclass	Abundance (%)
1	2,5-dimethyl-Pyrazine	Pyrazines	14.86
2	2,4-Di-tert-butylphenol	Phenylpropanes	8.69
3	2-Furanmethanol	Heteroaromatic compounds	5.77
4	methyl-Pyrazine	Pyrazines	5.47
5	2-ethyl-1-Hexanol	Fatty alcohols	3.36
6	2,6-dimethyl-Pyrazine	Pyrazines	2.90
7	3-methyl-1-Butanol	Alcohols and polyols	2.81
8	Pyrazine	Pyrazines	2.60
9	trimethyl-Pyrazine,	Pyrazines	1.30
10	3-methyl-Butanoic acid	Fatty acids and conjugates	1.00

## Data Availability

Data are contained within the article or Supplementary Materials.

## Supplementary Materials

The following supporting information can be downloaded at: https://www.mdpi.com/article/10.3390/jof11070538/s1, Table S1: GC-MS analysis of VOCs produced by R2.
